# Endogenous Intraocular Aspergillus Infection Associated With Systemic Vasculitis

**DOI:** 10.7759/cureus.40177

**Published:** 2023-06-09

**Authors:** Salil Mehta, Girish Soni

**Affiliations:** 1 Ophthalmology, Lilavati Hospital, Mumbai, IND; 2 Neurology, Lilavati Hospital, Mumbai, IND

**Keywords:** large-vessel vasculitis, voriconazole therapy, invasive fungal infections, retinal vasculitis, intraocular aspergillus

## Abstract

We report the systemic, ocular, and investigational findings of a 51-year-old male patient with large-vessel vasculitis and presumed ocular *Aspergillus* infection. He presented with persistent fever with left-sided weakness of the upper and lower limb for the past 15 days accompanied by profound visual loss in the left eye. A neurological examination revealed a left-sided ataxic hemiparesis with a significant reduction of power in both upper and lower limbs with dysarthria. He underwent neuroimaging which revealed a fresh non-hemorrhagic infarct in the left thalamocapsular and left parieto-occipital regions, suggestive of a stroke. A positron emission tomography/computed tomography scan revealed a diffuse low-grade uptake (standardized uptake value = 3.6) associated with a circumferential wall thickening involving the ascending aorta, arch of the aorta, and descending and abdominal aorta, suggestive of active large-vessel vasculitis. On examination, his visual acuity was 6/9 unaided in the right eye and perception of light with inaccurate projection in the left. A dilated fundus examination revealed multiple hemorrhages, cotton-wool spots, and areas of retinal thickening associated with a hard exudate in the right eye. A similar picture was seen in the left eye with the additional findings of a large (1 DD x 1 DD) subretinal whitish-yellowish mass with surrounding superficial retinal hemorrhages in the superior quadrant. A B-scan through the subretinal revealed non-visualization of the retinal pigment epithelium-Bruchs membrane layer with a large subretinal mass with a basal hyporeflective area and hyperreflective areas superiorly, suggesting a choroidal *Aspergillus* infection with infiltration of the overlying retina but without vitreous seeding. He was treated with anti-epileptics, oral and injectable blood thinners, oral antihypertensives, and oral antidiabetic medication. Intravenous methylprednisolone 1 g once daily was administered for five days, followed by oral prednisolone in tapering doses. In view of the ocular findings and the presumed diagnosis of ocular aspergillus, oral voriconazole 400 mg daily was added. At the last follow-up, the subretinal mass had completely resolved with a residual area of pigmentary degeneration with loss of retinal layer differentiation on the B-scan. There was also a marked reduction in the hemorrhages and cotton-wool spots in either eye, suggesting a marked improvement of the retinal vasculitis. A larger dataset would be needed to confirm a potential causative role for systemic fungal infections in large-vessel vasculitis.

## Introduction

Ocular fungal infections are a commonly encountered cause of visual morbidity and may potentially involve external structures such as the cornea, eyelids, or the internal tissues of the eye including the retina and choroid. Common fungi include *Candida *spp, *Aspergillus *spp., and *Mucormycosis *spp. *Aspergillus *is a highly prevalent saprophyte fungus, with infections commonly involving the lungs and sinuses [1].

*Aspergillus *intraocular infection produces panuveitis or endophthalmitis with highly significant ocular morbidity. Endophthalmitis, when it occurs after trauma, intraocular surgery, or following corneal infections, is termed exogenous and is usually encountered after cataract surgery. Endophthalmitis without an antecedent intraocular surgery or trauma is termed endogenous and is largely associated with an immunocompromised state such as that induced by human immunodeficiency virus (HIV) infection or following long-term immunosuppressive treatment and is due to hematogenous dissemination [2].

We report the systemic, ocular, and investigational findings of a 51-year-old male patient with magnetic resonance imaging (MRI) and positron emission tomography/computed tomography (PET/CT) findings suggestive of large-vessel vasculitis and an ocular examination suggestive of extensive retinal vasculitis and simultaneous intraocular aspergillus infection.

## Case presentation

A 51-year-old male patient presented with persistent fever and left-sided weakness involving both the upper and lower limb for the past 15 days accompanied by slurring of speech and profound visual loss in the left eye. He was a known case of diabetes mellitus (type 2) and systemic hypertension. There was no history/symptoms suggestive of COVID-19 infection and he had been vaccinated (two doses).

Significant past history included episodes of vertigo with difficulties in walking and sitting for 15 days. He had recently been treated (14 days earlier) for a cerebrovascular accident and had been discharged on a combination of oral clopidogrel 150 mg with aspirin 75 mg once a day, a combination of oral telmisartan 40 mg with metoprolol succinate 25 mg twice a day, oral prazosin 5 mg once a day, oral atorvastatin 40 mg once daily, oral cilnidipine 20 mg twice daily, and oral glimepiride 1 mg twice a day.

On systemic examination, he was conscious and oriented with normal vital parameters (pulse: 60 beats/minute; blood pressure: 130/80 mmHg; respiratory rate: 18 breaths/minute). Examination of the cardiovascular and respiratory systems was within normal limits. A neurological examination revealed a left-sided ataxic hemiparesis with a significant reduction of power in both upper and lower limbs with dysarthria.

**Figure 1:**
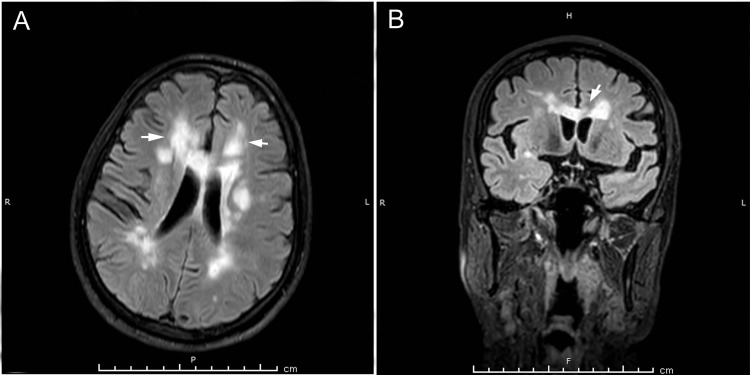
(A) Fluid attenuated inversion recovery (FLAIR) axial scan shows multiple FLAIR hyperintense signal intensities (demarcated by white arrows) in the corona radiata (bilaterally) and in the periventricular regions. (B) FLAIR coronal scan shows multiple FLAIR hyperintense signal intensities (demarcated by a white arrow) in the corona radiata (bilaterally) and in the periventricular regions.

Based on a clinical diagnosis of stroke, he underwent neuroimaging (MRI brain with gadolinium with MR angiography of the brain) which revealed a fresh non-hemorrhagic infarct in the left thalamocapsular and left parieto-occipital regions (Figures 1A, 1B). Chronic microhemorrhages were seen bilaterally in the cerebral hemispheres, thalami, brain stem, and cerebellum (bilaterally). Flattening of the carotid bulb and irregular narrowing of the internal carotid artery bilaterally and the M1 branch of the left middle cerebral artery were noted. All findings were suggestive of a stroke.

**Figure 2:**
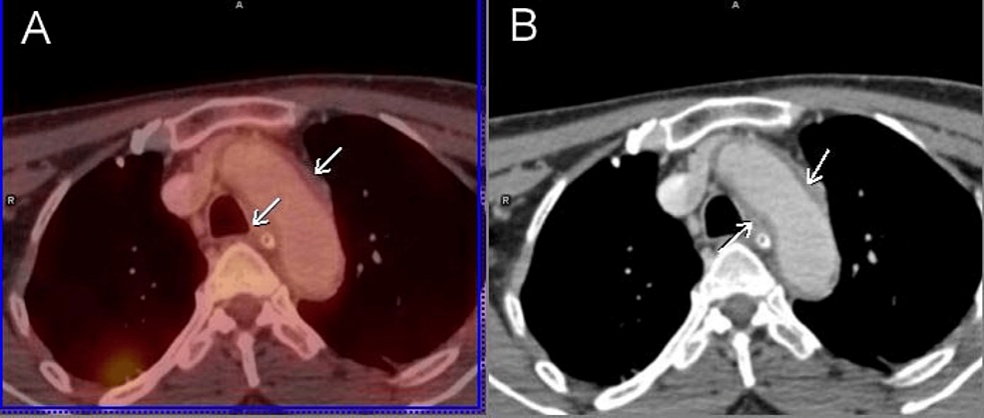
(A) Positron emission tomography/computed tomography (PET/CT) image showing thickening of the vessel wall of the aortic arch with diffuse low-grade uptake (white arrows). (B) Corresponding CT image showing vessel wall thickening (white arrows).

A PET/CT scan was administered to the patient with the use of 370 MBq of 18 fluorodeoxyglucose (FDG). Concomitantly, non-ionic intravenous contrast was administered. Areas of abnormal FDG uptake included scattered areas of reduced uptake in the entire brain more prominently in the parietal, medial temporal basal ganglia, and cerebellar regions. A diffuse low-grade uptake (standardized uptake value = 3.6) was associated with a circumferential wall thickening involving the ascending aorta, arch of the aorta, and descending and abdominal aorta (Figure 2A). Similar findings were also seen in the common iliac arteries bilaterally, right brachiocephalic, bilateral subclavian, and bilateral common carotid arteries. These findings were suggestive of active large-vessel vasculitis. The baseline CT scan showed evidence of wall thickening in these areas (Figure 2B).

The significant laboratory investigations are summarized below in Table 1.

**Table 1:** The relevant investigational findings.

Test name	Value	Normal range	Interpretation
Hemoglobin	10.60 g/dL	13.0–17.0 g/dL	Anemia
Total leucocyte count	13.64 × 1,000/mm^3^	4.0-10.00 × 1,000/mm^3^	Leucocytosis
Platelet count	494 × 1,000/mm^3^	150.0–410.0 × 1,000/mm^3^	Thrombocytosis
Blood urea nitrogen	26.0 mg/dL	6.0–20.0 mg/dL	Elevated
Serum creatinine	1.42 mg/dL	0.70–1.20 mg/dL	Elevated
Aspartate aminotransferase	79.80 U/L	0–40 U/L	Elevated
Alanine aminotransferase	129.0 U/L	<41 U/L	Elevated
Gamma-glutamyl transferase	183.0 U/L	8.0–61.0 U/L	Elevated
Erythrocyte sedimentation rate	112.0 mm	0–10 mm	Elevated
C-reactive protein	111.0 mg/L	<5.0 mg/L	Elevated
Anti-nuclear antibodies	Negative	Negative	Negative
Anti-PR3 (c-ANCA)	Negative	Negative	Negative
Anti-MPO (p-ANCA)	Negative	Negative	Negative
Glycosylated hemoglobin	7.7%	<5.6%	Elevated
Serum galactomannan	1.36 Units	<0.50 negative, >0.50 positive	Positive
Serum 1,3-BD flucan	<7.0 pg/mL	<60 pg/mL negative	Negative
Serum D-dimer	228 ng/mL	0.0–243 ng/mL	Negative

The investigations suggested a pro-inflammatory state, as noted by the elevated leucocyte count, C-reactive proteins, and erythrocyte sedimentation rate. A baseline search for autoimmune antibodies was negative but the MRI and PET/CT investigational findings suggested extensive systemic vasculitis. The patient had mildly deranged renal and hepatic functions as well. A joint rheumatology/neurology consult evaluated these findings and agreed on a diagnosis of active large-vessel vasculitis with the need for early intravenous pulse steroid therapy.

**Figure 3:**
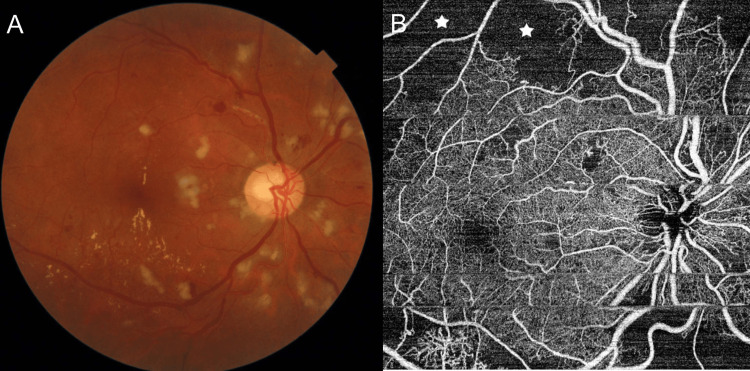
(A) Fundus photo of the right eye with extensive hemorrhages, cotton-wool spots, and hard exudates. (B) Optical coherence tomography angiography showing extensive areas of capillary non-perfusion (white stars).

**Figure 4:**
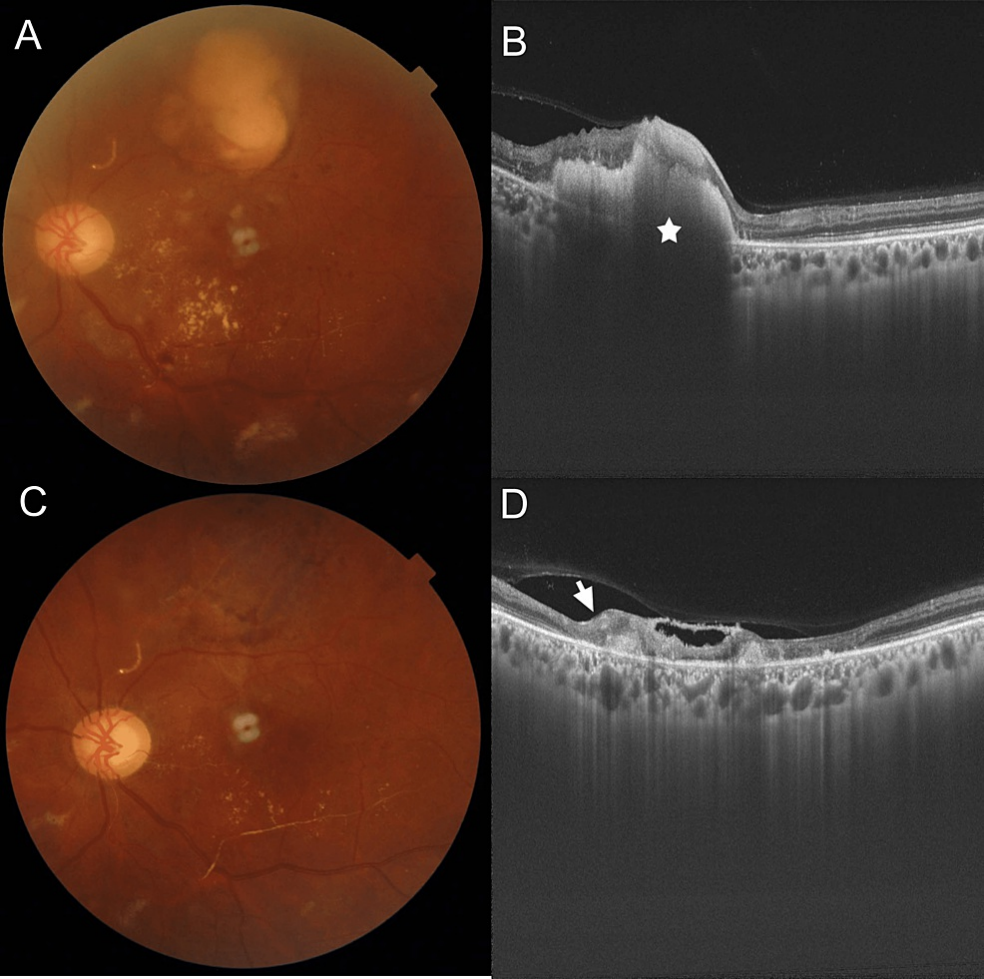
(A) A fundus photo of the left eye showing multiple hemorrhages, cotton-wool spots, vasculitis, and areas of retinal thickening associated with hard exudates. There is a large (1 DD × 1 DD) subretinal whitish-yellowish mass with surrounding superficial retinal hemorrhages in the superior quadrant. (B) A B-scan reveals non-visualization of the retinal pigment epithelium-Bruchs membrane layer with a large subretinal mass with a basal hyporeflective area and hyperreflective areas superiorly, suggesting a choroidal nidus of Aspergillus infection with infiltration of the overlying retina but without vitreous seeding. (C) On the last follow-up, the subretinal mass had completely resolved with a residual area of pigmentary degeneration with thinning. (D) A B-scan shows thinning and loss of retinal layer differentiation.

Subsequently, he was referred to the ophthalmology department for an evaluation of his left-sided visual loss. On examination, his visual acuity was 6/9 unaided in the right eye and perception of light with inaccurate projection in the left. An anterior-segment evaluation of either eye was unremarkable except for a relative afferent pupillary defect of the left eye. A dilated fundus examination revealed multiple hemorrhages, cotton-wool spots, and areas of retinal thickening associated with hard exudate in the right eye (Figure 3A). An optical coherence tomography angiography (OCTA) scan in the right eye revealed extensive areas of superficial capillary plexus loss consistent with extensive vasculitis (Figure 3B). A similar picture was seen in the left eye with the additional findings of a large (1 DD × 1 DD) subretinal whitish-yellowish mass with surrounding superficial retinal hemorrhages in the superior quadrant (Figure 4A). An OCTA scan in the left eye was non-contributory due to persistent fixation difficulties. A B-scan through the subretinal lesion was possible and revealed non-visualization of the retinal pigment epithelium-Bruchs membrane layer with a large subretinal mass with a basal hyporeflective area and hyperreflective areas superiorly, suggesting a choroidal breakthrough of *Aspergillus *infection with infiltration of the overlying retina but without vitreous seeding. (Figure 4B). A positive result for serum galactomannan suggested a diagnosis of invasive fungal infection specifically *Aspergillus* spp.

He was treated with anti-epileptics (oral levetiracetam 500 mg twice a day), oral aspirin 75 mg once a day, oral atorvastatin 20 mg once a day, oral antihypertensives (cilnidipine 20 mg twice daily, oral telmisartan 40 mg with metoprolol succinate 25 mg twice a day, oral prazosin 5 mg once a day), injectable enoxaparin 40 mg twice a day, injectable piracetam 1 g twice daily, oral modafinil 100 mg once daily, and oral glimepiride 1 mg twice a day. Intravenous methylprednisolone 1 g once daily was administered for five days, followed by oral prednisolone (50 mg/day) once a day in tapering doses (10 mg weekly). In view of the ocular findings and the presumed diagnosis of ocular *Aspergillus*, oral voriconazole 400 mg twice a day for one day followed by 200 mg twice a day was added.

At the first follow-up (six weeks), there was a significant reduction of the subretinal mass which was corroborated by a reduction in the mass size on the B-scan. At the last follow-up, the subretinal mass had completely resolved with a residual area of pigmentary degeneration (Figure 4C) with loss of retinal layer differentiation on the B-scan (Figure 4D). There was also a marked reduction in the hemorrhages and cotton-wool spots in either eye suggesting a marked improvement of the retinal vasculitis. The best-corrected vision was 6/9, N8 in the right, but was unchanged in the left. There was a significant improvement in his systemic status with marked improvement in the muscle power of his hemiplegic side and dysarthria.

## Discussion

We report the clinical and investigational findings of a middle-aged male with MRI and PET/CT-proven large-vessel vasculitis associated with a presumed ocular aspergillus infection and its successful treatment with a combination of oral steroids and voriconazole.

Ocular fungal infections are an important cause of ocular morbidity and may involve various tissues including the external structures such as the cornea, eyelids, lacrimal apparatus, or internal tissues including the retina and choroid. Common causative organisms include *Candida *spp, *Aspergillus *spp., and *Mucormycosis* spp. *Aspergillus* is an innocuous, highly prevalent saprophyte fungus, and its infections commonly involve the lungs and sinuses. Commonly encountered species include *Aspergillus fumigatus*, *Aspergillus flavus*, *Aspergillus niger*, and *Aspergillus terreus*.

*Aspergillus *spp. may involve the external tissues of the eye, commonly the cornea, but may also cause conjunctivitis and lacrimal system infections. The ingress of *Aspergillus *infection into the eye produces panuveitis or endophthalmitis with highly significant ocular morbidity. Exogenous endophthalmitis usually occurs after trauma, intraocular surgery, or following corneal infections. Endophthalmitis without an antecedent intraocular surgery or trauma is termed endogenous and is largely associated with an immunocompromised state such as that induced by HIV infection, malignancy, following long-term immunosuppressive treatment, and is due to hematogenous dissemination from a systemic focus of infection [1,2].

Rarely, immunocompetent individuals with no previous risk factors may also be affected by *Aspergillus*-induced endophthalmitis. Logan et al. reported the clinical findings and management of a 78-year-old lady with uniocular visual loss. She was on a short course of steroids for possible giant-cell arteritis and developed panuveitis over a period of five days. A vitreous biopsy and subsequent culture on Sabourauds agar revealed *Aspergillus fumigatus* and she was initiated on oral voriconazole 400 mg twice a day for 24 hours, followed by a maintenance treatment at a dose of 200 mg twice a day for a two-week course for complete resolution [3]. Rana et al. reported a case of *Aspergillus fumigatus* endophthalmitis following an aortic root replacement eight months earlier [4]. A literature search on PubMed from 1991 to 2022 using the keywords “Aspergillus AND endophthalmitis AND immunocompetent” and “Aspergillus AND ocular AND immunocompetent” returned 29 papers. We screened these to detect cases without previous intraocular surgery, trauma, or immunosuppressive treatment. In total, 15 papers fit these criteria and described the findings of 14 patients with endophthalmitis/panuveitis, and one paper described a patient with *Aspergillus *iris granuloma. Some of these patients, however, reported intravenous infusions a few days before the onset of symptoms or pre-existing pulmonary disease with presumed *Aspergillus *infection [3-17].

There is a complex interaction between systemic infections and vasculitis. A large body of research has linked Takayasu arteritis with *Mycobacterium tuberculosis*, giant cell arteritis with *Burkholderia*, and polyarteritis nodosa with hepatitis B virus, hepatitis C virus, and HIV infections. Similarly, the role of *Aspergillus* spp. as a causative agent of cerebral vasculitis and infarction is increasingly being recognized. Roberts et al. reported the case of a 71-year-old female who was treated for a history of malaise and headaches. Post-mortem findings included a thrombosis of the basilar artery, with extensive infiltration of the wall with the characteristic branching septate hyphae [18]. Haddad et al. described the pathological findings of three patients with proven invasive cerebral aspergillosis who presented clinically with diffuse vasculitis involving the large caliber cerebral vessels [19].

Possible mechanisms of interplay between infection and immunity include direct mechanisms such as hematogenous septic emboli or direct invasion of the blood vessel walls. Indirect mechanisms include (A) activation of T and B cells: infections may stimulate an autoimmune response due to shared epitopes between pathogens and host or upregulated heat shock proteins. Circulating toxins may act as superantigens and these could induce oligoclonal activation of T cells. (B) Immune complex-mediated damage: this represents a type III hypersensitivity where the infectious organisms act as antigens. These immune complexes are lodged within vessel walls and stimulate an immune response. (C) cell-mediated hypersensitivity type IV: exposure to antigens may attract lymphocytes which release cytokines that cause further inflammatory cell activation and tissue damage [20].

The patient we report had large-vessel vasculitis with presumed ocular *Aspergillus *infection. The diagnosis was based on a positive serum galactomannan test, and the therapeutic response to voriconazole negated the need for a diagnostic vitrectomy with the attendant risks but we agree a diagnostic vitrectomy would have been confirmatory and allowed species identification as well. The fundus/OCT picture at diagnosis was non-specific for diagnosing retinal vasculitis and there is a possibility of concurrent diabetic retinopathy which also presents with cotton-wool spots/hemorrhages. The OCT showed extensive capillary non-perfusion which is a feature of both disease conditions. However, the marked improvement in the clinical picture with the resolution of the cotton-wool spots/hemorrhages suggests a significant vasculitic component that responded to steroid therapy. We opted not to perform a vitrectomy as we felt the clear vitreous without seeding would not permit any significant microbiological result. For similar reasons, we opted to treat this patient conservatively, with oral voriconazole and regular monitoring. The duration of therapy was determined by an infectious disease consult.

This patient had a combination of large-vessel vasculitis with ocular *Aspergillus *(presumably hematogenous) but it is not possible to confirm a causative role of the fungal infection for the large-vessel vasculitis, and the dual pathologies may simply represent an associated secondary infection due to diabetes mellitus or other undefined factors. A larger dataset would be needed to confirm a potential causative role of systemic fungal infections in large-vessel vasculitis.

## Conclusions

The patient we report presented with an autoimmune pathology in the form of large-vessel vasculitis which had its clinical correlates in the stroke/dysarthria with imaging correlations on the MRI and PET/CT scans. There was extensive retinal vasculitis possibly due similar pathogenic mechanism. This had its clinical correlates in the hemorrhages and cotton-wool spots which showed a significant improvement following steroid therapy. Similarly, there was marked systemic improvement.

Additionally, there was an associated intraocular infection presumably *Aspergillus *which correlated well with the B-scan and serological studies. The use of oral voriconazole led to a complete resolution with residual retinal atrophy. We highlight this dual pathology which may be a result of an unrelated secondary invasive fungal infection in a patient with diabetes mellitus or may suggest a causal role of the infection in the development of vasculitis. A larger dataset would be needed to study this possibility.
